# Patterns of Mitochondrial TSPO Binding in Cerebral Small Vessel Disease: An *in vivo* PET Study With Neuropathological Comparison

**DOI:** 10.3389/fneur.2020.541377

**Published:** 2020-10-16

**Authors:** Paul Wright, Mattia Veronese, Ndabezinhle Mazibuko, Federico E. Turkheimer, Eugenii A. Rabiner, Clive G. Ballard, Steven C. R. Williams, Avinash Kumar Hari Narayanan, Bahiya Osrah, Ricky Williams, Tiago R. Marques, Oliver D. Howes, Federico Roncaroli, Michael J. O'Sullivan

**Affiliations:** ^1^Department of Neuroimaging, Institute of Psychiatry Psychology & Neuroscience, King's College London, London, United Kingdom; ^2^Invicro, London, United Kingdom; ^3^College of Medicine and Health, University of Exeter, Exeter, United Kingdom; ^4^Division of Neuroscience and Experimental Psychology, Faculty of Biology, Medicine and Health, University of Manchester, Manchester, United Kingdom; ^5^Manchester Centre for Clinical Neuroscience, Salford Royal Foundation Trust, Salford, United Kingdom; ^6^Department of Psychosis Studies, Institute of Psychiatry, Psychology and Neuroscience, King's College London, London, United Kingdom; ^7^University of Queensland Centre for Clinical Research, Brisbane, QLD, Australia; ^8^Department of Neurology, The Royal Brisbane and Women's Hospital, Herston, QLD, Australia

**Keywords:** microglia, PET—positron emission tomography, TSPO (18 kda translocator protein), small vessel disease (SVD), immunohistochemistry

## Abstract

Small vessel disease (SVD) is associated with cognitive impairment in older age and be implicated in vascular dementia. Post-mortem studies show proliferation of activated microglia in the affected white matter. However, the role of inflammation in SVD pathogenesis is incompletely understood and better biomarkers are needed. We hypothesized that expression of the 18 kDa translocator protein (TSPO), a marker of microglial activation, would be higher in SVD. Positron emission tomography (PET) was performed with the second-generation TSPO ligand [^11^C]PBR28 in 11 participants with SVD. TSPO binding was evaluated by a two-tissue compartment model, with and without a vascular binding component, in white matter hyperintensities (WMH) and normal-appearing white matter (NAWM). In post-mortem tissue, in a separate cohort of individuals with SVD, immunohistochemistry was performed for TSPO and a pan-microglial marker Iba1. Kinetic modeling showed reduced tracer volume and blood volume fraction in WMH compared with NAWM, but a significant increase in vascular binding. Vascular [^11^C]PBR28 binding was also increased compared with normal-appearing white matter of healthy participants free of SVD. Immunohistochemistry showed a diffuse increase in microglial staining (with Iba1) in sampled tissue in SVD compared with control samples, but with only a subset of microglia staining positively for TSPO. Intense TSPO staining was observed in the vicinity of damaged small blood vessels, which included perivascular macrophages. The results suggest an altered phenotype of activated microglia, with reduced TSPO expression, in the areas of greatest white matter ischemia in SVD, with implications for the interpretation of TSPO PET studies in older individuals or those with vascular risk factors.

## Introduction

Cerebrovascular small vessel disease (SVD) is increasingly recognized as an important cause of age-related cognitive impairment and dementia. The term defines a heterogeneous group of hereditary and sporadic conditions that impair the brain microcirculation (1). Neuropathological findings in SVD affect parenchymal and leptomeningeal small perforating arteries, arterioles, capillaries and, less commonly, small veins, and venules (2). The radiological manifestations include focal lacunar infarcts and ill-defined lesions, hyperintense on T2-weighted MRI. These abnormalities occur in both deep white matter, with relative sparing of subcortical U-fibers, and the deep gray matter of the thalamus and striatum. Diffuse lesions in white matter are often referred to as diffuse white matter hyperintensities (WMH) or leukoaraiosis (3). Histologically, diffuse lesions correspond to areas of rarefaction of myelin (4), enlargement of perivascular spaces but general sparing of the neuropil. Hallmark pathology of small vessels in these regions includes endothelial proliferation, thickening and splitting of the walls, small plaque-like accumulations of plasma proteins, perivascular reactive astrocytosis, and accumulation of perivascular macrophages (5). Increasingly, interest has shifted toward a critical role for the neurovascular unit in the pathogenesis of SVD (6), with evidence for compromise of the blood-brain barrier (BBB) and cerebral blood flow regulation (7, 8). This has reignited interest in inflammation, as regulation of immune responses is a cardinal function of the neurovascular unit and blood-brain barrier.

Neuropathological evidence supports a role for inflammation in the pathogenesis of SVD. Activated microglia are abundant in areas of damaged white matter, more so than in areas of morphologically unaffected control white matter. Microglia cells show the morphology of activated cells, which suggests immune response and a role in antigen presentation, possibly in response to BBB disruption, and extravasation of plasma proteins (9). Epidemiological evidence also suggests an association between systemic inflammation and WMH extent (10). Microglial activation is associated with increased metabolic activity and mitochondrial biogenesis. The latter leads to enhanced expression of the mitochondrial 18 kDa translocator protein (TSPO) (11). This alteration in protein expression provides a possible molecular marker of microglial activation, which has been exploited by positron emission tomography (PET) studies, using radioligands to investigate microglial activation *in vivo* in the human brain. PET studies in a number of neurodegenerative and neuroinflammatory diseases have shown higher TSPO expression, consistent with *post-mortem* studies showing microglial activation (12). Moreover, PET imaging studies suggest that focal lacunar infarction, a feature of SVD, initiates microglial activation (13). However, few data are available on TSPO in the diffuse, progressive form of SVD (14). The role of inflammation in diffuse SVD has important therapeutic implications, as WMH progression is associated with cognitive decline and risk of dementia. New treatment avenues to retard progression of diffuse SVD are therefore urgently needed.

This study utilized the second generation (15) tracer [^11^C]PBR28 to investigate TSPO binding patterns in individuals with SVD. Based on *post-mortem* observations, we hypothesized that [^11^C]PBR28 binding would be higher within WMH than normal-appearing white matter (NAWM). We included a group of participants with symptomatic lacunar stroke within the last 12 months, to investigate whether acute infarction modulated inflammation within SVD. We compared these results with a dataset of [^11^C]PBR28 brain PET scans from healthy controls. To facilitate interpretation of PET binding results, we also investigated *post-mortem* brains of subjects with neuropathologically-confirmed SVD.

## Methods

### SVD Participants

SVD was defined as the presence of confluent or near confluent (corresponding to Fazekas grade 2 or above) WMH on T2-weighted MRI scans of the brain. Participants were defined as “asymptomatic” if they had no history of stroke or TIA and were free of cognitive and gait symptoms. Asymptomatic SVD participants were recruited from the local community. Symptomatic SVD patients (individuals with a history of lacunar stroke in the last year) were recruited from a cohort enrolled in the longitudinal STRATEGIC study of cognitive function after stroke (registered with ClinicalTrials.gov as https://www.clinicaltrials.gov/show/NCT03982147). All participants were aged over 50 and fluent in English; we excluded those with large artery infarcts, diagnosis of dementia, active malignancy, major neurological, or psychiatric illness (as defined by DSM-IV-TR), previous moderate to severe head injury (Mayo clinic classification of severity) or lack of capacity to consent. Those invited to participate in the PET study all had high or medium TSPO binding status based on Ala147Thr polymorphism genotyping (4, 16). In total, six asymptomatic SVD participants and five stroke patients underwent PET (Table 1).

**Table 1:** Participant demographics.

**Group**	**ID**	**Age at scan/death**	**Sex**	**Extent of white matter lesions**	**Clinical diagnosis**	**TSPO tracer affinity**	**Tau Braak stage**	**APOE**
PET asymptomatic SVD	P1	73	F	Fazekas DWM grade 2	Healthy	High	n/a	n/a
	P2	78	F	Fazekas DWM grade 2	Healthy	High	n/a	n/a
	P3	85	F	Fazekas DWM grade 2	Healthy	Mixed	n/a	n/a
	P4	69	F	Fazekas DWM grade 2	Healthy	High	n/a	n/a
	P5	90	M	Fazekas DWM grade 2	Healthy	High	n/a	n/a
	P6	72	F	Fazekas DWM grade 3	Healthy	High	n/a	n/a
PET symptomatic SVD	P7	58	M	Fazekas DWM grade 2	Lacunar stroke	High	n/a	n/a
	P8	86	M	Fazekas DWM grade 2	Lacunar stroke	High	n/a	n/a
	P9	51	M	Fazekas DWM grade 3	Lacunar stroke	High	n/a	n/a
	P10	79	M	Fazekas DWM grade 2	Lacunar stroke	Mixed	n/a	n/a
	P11	55	M	Fazekas DWM grade 2	Lacunar stroke	High	n/a	n/a
PET controls	*N* = 21	*M* 37.8 *SD* 15.7	6 female 14 male	Fazekas DWM grade 0 No striatal lacunes	Healthy	3 mixed 18 high	n/a	n/a
Post-mortem controls	PDC013	77	M	Mild aging changes	Aging	n/a	II	n/a
	C073	77	M	Mild aging changes	Hepatocarcinoma	n/a	II	n/a
Post-mortem patients	DPM10/24	92	F	Severe SVD	Vascular dementia	n/a	III	ε3ε3
	DPM16/07	71	F	Severe SVD	Aging	n/a	I	ε3ε3
	DPM16/19	97	F	Severe SVD with microinfarction	Aging	n/a	II	ε3ε3
	DPM16/15	92	F	Severe SVD with ischemic lesions	Vascular dementia	n/a	II	ε3ε4
	DPM16/02	90	M	Severe SVD with microinfarctions	Aging	n/a	II	ε3ε3

The study was approved by the Bromley Research Ethics Committee (ref: 13-LO-1745) and was conducted in accordance with the Declaration of Helsinki. All participants provided written, informed consent.

### [^11^C]PBR28 PET Imaging

Radiopharmaceutical preparation of [^11^C]PBR28 was performed as previously described (15) and the imaging protocol was adopted from previous [^11^C]PBR28 PET studies (17). Briefly, an initial low-dose computed tomography (CT) scan was acquired for attenuation and scatter correction using a Siemens Biograph™ True Point™ PET/CT scanner (Siemens Medical Systems, Germany). Subjects then received a bolus injection of [^11^C]PBR28 (injected dose mean ± SD 349 ± 10 MBq) followed by a 90-min PET emission scan. PET data were acquired in three-dimensional mode and binned into 26 frames (durations: 8 × 15 s, 3 × 1 min, 5 × 2 min, 5 × 5 min, 5 × 10 min). Images were corrected for attenuation and scatter and reconstructed using filtered back projection.

In parallel to the PET acquisition, arterial blood was sampled from the radial artery using a combined automatic (from 0 to 15 min after tracer injection) and manual approach (samples collected at 5, 10, 15, 20, 25, 30, 40, 50, 60, 70, 80, and 90 min) in agreement with the experimental protocol used in previous publication (18). From these blood samples, a time-continuous metabolite-free plasma input function was derived for each individual to describe the tracer delivery to the brain by using Multiblood software (https://github.com/MatteoTonietto/MultiBlood) (19). PET scans started at a similar time of day to reduce potential effects of circadian rhythm on TSPO density (between 10.00 a.m. and 3.30 p.m.) (20). Cumulative scanner movement, defined as the sum of total frame-to-frame movement during imaging acquisition, was 15.2 ± 4.6 (mean ± SD) mm and none of the participants showed interframe motion spikes >5 mm. Free plasma fraction (*f*_*p*_) ranged from 1.4 to 2.7% (mean ± SD: 1.8 ± 0.4%). These numbers are qualitatively comparable with the historical archive of dynamic [^11^C]PBR28 PET studies (17, 18, 21).

### Magnetic Resonance Imaging (MRI)

MRI sequences were acquired using an MR750 3.0 Tesla MR scanner (GE Healthcare, Little Chalfont, Buckinghamshire, United Kingdom). We selected sequences to meet the STandards for ReportIng Vascular changes on nEuroimaging (STRIVE) (22). T1-weighted scans were acquired with Magnetization Prepared Rapid Gradient Echo (MPRAGE) sequence with repetition time of 7.312 ms, echo time of 3.016 ms and a flip angle of 11°. Images were acquired in the sagittal plane covering the whole head with field of view (FOV) of 270 × 270 mm and matrix size of 256 × 256 voxels. Slice thickness and slice gap were 1.2 mm. T2-weighted fast recovery fast spin echo (FRFSE) and fluid-attenuated inversion recovery (FLAIR) sequences were acquired to delineate infarcts and other vascular lesions. The FRFSE sequence used a TR of 4,380 ms, TE of 54–65 ms and flip angle of 90–111°. The FLAIR sequence used a TR of 8,000 ms, TE of 120–130 ms and flip angle of 90–111°. Images were acquired in the axial plane with FOV of 240 × 240 mm and matrix sizes of 320 × 256 and 256 × 128 voxels for FRFSE and FLAIR, respectively. Slice positions were aligned for both sequences with 72 slices at 2 mm thickness for FRFSE and 36 slices at 4 mm thickness for FLAIR. Perfusion measurements were obtained from an arterial spin labeling acquisition. Pseudo-continuous arterial spin labeling (ASL) MRI was obtained (geometry of 1.875 × 1.875 × 3 mm and a post-labeling delay of 2,525 ms). One patient (P2) was unable to have a research MRI scan and clinical MR images obtained at 1.5T with a comparable FLAIR acquisition were used to define WMH. An additional patient did not have ASL during their research scan, leaving nine out of eleven patients with ASL scans.

### Image Processing and Analysis

All PET images were corrected for head movement by realigning all the PET frames to a single “reference” space identified by the PET frame with the highest activity as implemented in MIAKAT^TM^ (http://www.miakat.org). Regional time-activity curves were obtained by sampling the dynamic PET image with predefined regions of interest (ROIs). ROIs delineating WMH were drawn manually on FLAIR images by a single rater (PW). White matter was defined using automated segmentation of T1-weighted images in SPM12. NAWM ROIs were defined by subtracting WMH voxels from segmented white matter. For comparison with the post-mortem tissue analysis (see below), a striatum ROI was defined using the Hammersmith atlas, combining the caudate, putamen and nucleus accumbens (23).

**Figure 1:**
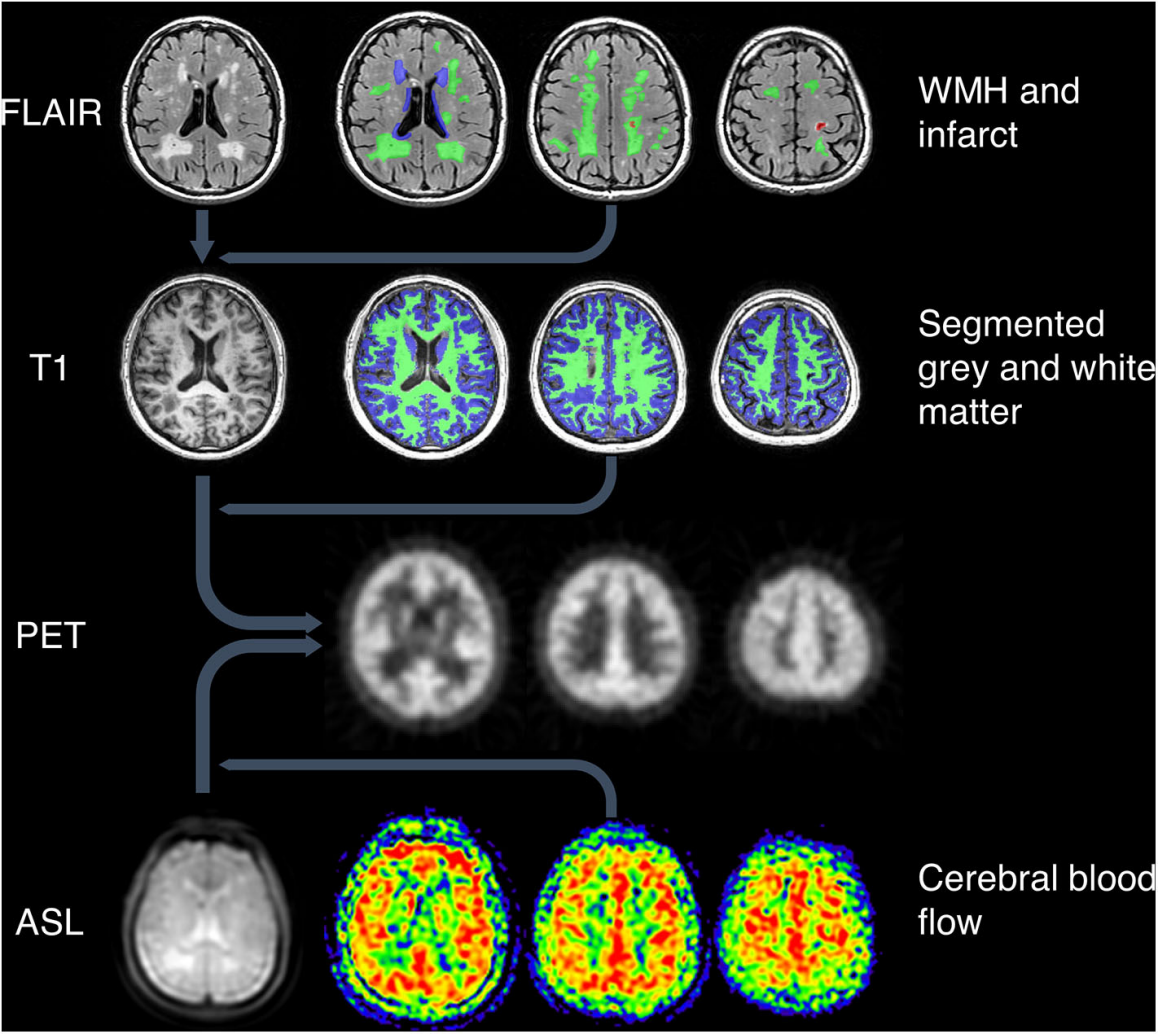
Image processing pipeline. Top: WMH and infarcts were drawn on T2-weighted FLAIR images, which were co-registered with T1-weighted images along with the ROIs. Green = deep WMH. Blue = periventricular WMH. Red = infarct lesion. Second row: grey and white matter were segmented using T1-weighted images. Green = white matter. Blue = gray matter. All MRI images and ROIs were then co-registered to PET space (third row). Bottom row: ASL proton density images (left image) were co-registered to PET space along with CBF maps (right three images).

MR images and their ROIs were co-registered with a reference PET image for each subject to allow analysis of tracer uptake within specified ROIs (Figure 1). Using SPM12 (Functional Imaging Laboratory, University College London, UK), the FLAIR images were co-registered with the high resolution T1-weighted images, which were in turn co-registered with the reference PET image. The ASL images were co-registered with the T1-weighted images in PET space.

ASL images were processed to generate maps of cerebral blood flow (CBF) and a matched proton density weighted image as described previously (24). The proton density images show anatomical features in the same geometry as CBF and were used for co-registration to PET space.

Quantification of [^11^C]PBR28 tissue distribution was performed using the standard 2-tissue compartmental modeling (2TCM) and 2-tissue compartmental model with the inclusion of vascular binding (2TCM-1K) (25). Both models have been used with [^11^C]PBR28 PET for TSPO tissue quantification in controls and patients (18, 26) and tested in TSPO specific blocking studies (15, 17). In a principally vascular disease, modeling the vascular compartment could provide useful disease-relevant information, therefore the 2TCM-1K approach had potential advantages for clinical application to SVD. Note that these kinetic models share most of the micro-parameters with the exception of *K*_*b*_ which is explicitly used in 2TCM-1K to account for the vascular TSPO component. Inclusion of an explicit vascular compartment allows the model to account for two possible confounding factors in SVD: reduced tissue blood flow; and binding of TSPO by the endothelium (so that the tracer remains within the vascular compartment). Both blood flow and altered blood-to-tissue extraction are modeled by the *K*_1_ parameter. In addition to *K*_*b*_, the total volume of distribution of the tracer in tissue (*V*_*T*_, ml/cm^3^), the blood-to-tissue tracer transport constant (*K*_1_, ml/g/cm^3^), and the blood-to-tissue volume fraction (*V*_*b*_, no units) were considered main parameters of interest. A full description of the model kinetic parameters and their mathematical identifiability is reported in original references (18).

### Healthy Control Participants

A dataset of [^11^C]PBR28 brain PET scans from healthy individuals was obtained from an institutional PET repository NODE (Maudsley Biomedical Research Center, London, UK). This dataset was used to compare the [^11^C]PBR28 PET signal in the NAWM and WMH of the SVD participants with healthy WM tissue. Twenty-one healthy controls (age: 38 ± 15 years, gender: 15 males/6 females, 3 MABs/18 HABs) were included in this analysis. Radiotracer synthesis, experimental protocol, image acquisition and reconstruction, analysis pipeline, and software were consistent across cohorts (18). To confirm that the control participants were free of SVD, T1-weighted MR images were inspected, to confirm the absence of diffuse white matter hypointensities or focal lesions >4 mm in the basal ganglia (4, 27).

### Post-mortem Tissue Analysis

Five *post-mortem* brains found to contain neuropathological evidence of sporadic SVD, were selected from the Manchester Brain Bank cohort (ethics: 09/H0906/52+5). The subjects' ages ranged between 71 and 97 years; four were female. The individual samples were selected for relatively pure vascular pathology: all had low levels of tau-related pathology consistent with Braak & Braak stage II and low amyloid load (Thal phase III) (28–30). *APOE* allele was ε3/ε3 in all subjects. None of the brains demonstrated amyloid angiopathy. Cases with watershed infarcts and laminar necrosis were excluded. From the coronal slices stored in 10% buffered formalin, we resampled the striatum at the levels of the septum and nucleus accumbens, and at the level of anterior commissure. The demographics and essential clinical information of the five subjects are reported in Table 1.

Two control brains from individuals of comparable age were supplied by the UK Parkinson's Society Brain Bank at Imperial College, London, UK (ethics: 18/WA/0238). They were selected from a cohort of over 900 brains using the following stringent criteria: mild SVD, absence of α-synuclein and TDP-43 inclusions, tau Braak stage I–III, and low Thal Aβ phase. None of the subjects had previous history of stroke or cerebrovascular disease. Post-mortem delay for all cases was <48 h. Similar to SVD cases, samples of the basal ganglia were taken at the levels of septum and anterior commissure. The H&E-stained sections were reviewed to confirm the diagnosis; immunostains for α-synuclein, Tau, Aβ peptide, p62, and TDP-43 were also available for review. The two control donors were males aged 71 and 77 years at the time of death. The cause of death was malignancy in both cases; brain metastases were not present (Table 1).

### Tissue Analysis and Quantification of Microglia/Macrophage Density and TSPO Expression

The tissue samples were routinely processed over 3 days on a Shandon Citadel 1000 Processor and embedded in molten Histoplast Paraffin wax using a ThermoFisher HistoStar embedding station. Ten consecutive sections number from 1 to 10 were cut at 5 μm from each block on a Shandon Finesse 325 microtome. Section Introduction was stained with hematoxylin-eosin (H&E). Sections Methods and Results were used for immunohistochemistry. Dewaxing, rehydration and antigen retrieval was performed using a Ventana BenchMark Ultra and the reagents supplied by the manufacturer as per the standard preprogramed protocol (Ventana Medical Systems, Roche Group, Tucson, AZ, USA). The anti-ionized calcium binding adaptor molecule 1 (Iba1) polyclonal antibody (Wako, 019-19741) for microglia and macrophages was used at the dilution of 1:5,000 and incubated for 32 min. The anti-TSPO polyclonal antibody (Abnova, PAB7095) was used at the dilution of 1:250 for 60 min. Nuclei were counterstained with hematoxylin and both post-counterstained in bluing reagent for 4 min.

### Quantification of Iba1 and TSPO Immunostains

Immunostains were scanned at the Bioimaging Facility at University of Manchester (www.bmh.manchester.ac.uk/research/facilities/bioimaging) using 3D Histech Pannoramic 250 slide scanner (3D Histech ltd, Hungary). Anatomical landmarks were outlined on the digital images and region of interest for quantification were randomly chosen by the 3D Histech program. The regions were selected in the head of caudate, anterior and posterior putamen, globus pallidus, and anterior and posterior limbs of the internal capsule. Iba1 and TSPO positive microglia were counted at the magnification of x20 in 60 ROIs overall in each SVD case and control brain. The accuracy of anatomical margins was validated by an experienced neuropathologist (FR). All images from ROIs were then imported to ImageJ using the Kurt De Vos cell counter (https://imagej.NIH.gov/ij, USA) for post-production editing and evaluation.

Two separate automated counting macros coded in JavaScript were run on ImageJ to count the percentage of area occupied by positive staining signal of TSPO or Iba1 in each ROI (see Supplementary Material). Each image was separated into the three RGB channels by applying the Color Deconvolution tool using the H DAB vector. The red channel, showing the oxidized DAB brown precipitate indicating positive immunohistochemistry staining for TSPO or Iba1, was selected and converted to a binary image. The threshold was adjusted to minimize background staining artifacts and applied consistently across each ROI. The percentage area occupied by the positively stained signal indicated by black was calculated with the Analyze Particle tool (Supplementary Figure 1). Each of the two automated counting macros for TSPO and Iba1 were applied uniformly across all ROI following a quality control assessment comparing manual counts with the automated macros for each stain. Double blinded validation was also carried out independently by three co-authors (RW, BO, and FR) with multipoint tagging on ImageJ. Only microglial cells with a clearly identifiable nucleus were counted.

### Statistical Analysis

Statistical analyses were performed using SPSS Statistics version 25 (IBM UK Ltd., Portsmouth, UK). For the imaging data analysis, differences in kinetic parameter estimates between the modeled ROIs were tested using paired *t*-tests. Comparisons between groups were tested using independent sample *t*-tests. Equality of variances was tested using Levene's test and correction applied to the degrees of freedom for comparisons where this assumption was not met. Data points were excluded if the values were extreme (more than three interquartile ranges from the edge of the interquartile range) or physiologically implausible (*K*_*b*_ values >1 or close to 0 min^−1^). Exclusions were made on a by-ROI basis, with data points for all parameters for a given ROI being excluded if one parameter was invalid. For the post-mortem study, the effect of SVD on microglia/macrophage density and Iba1 expression was tested using two-way analysis of variance (ANOVA). Given the low number of subjects, each region was considered a separate measure, with region and group as fixed effects.

## Results

### TSPO PET Binding in Lesions and NAWM

The main PET findings were consistent between 2TCM-1K and 2TCM models. Therefore, unless otherwise stated, the results below refer to 2TCM-1K (for a full comparison with the 2TCM, please see Supplementary Material).

**Figure 2:**
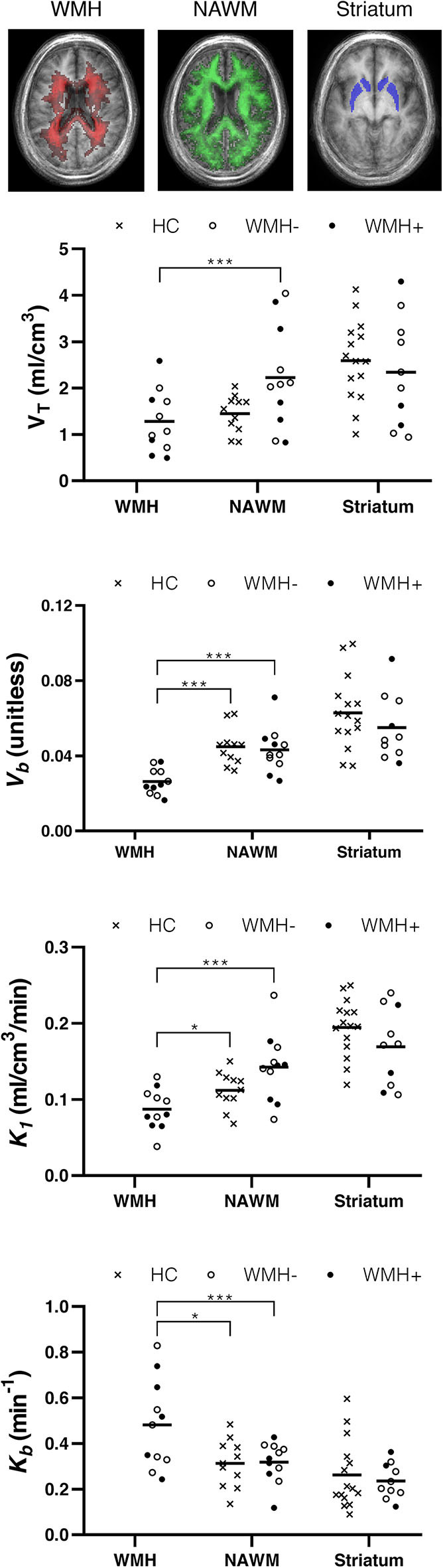
TSPO binding differs between normal appearing white matter (NAWM) and white matter hyperintensities (WMH). Top row: group frequency maps of WMH (red) and NAWM (green) with voxel intensity indicating number of participants with corresponding tissue type, and the atlas region defining the striatum (blue). Plots show volume of tracer (*V*_*T*_), tissue-to-blood ratio (*V*_*b*_), plasma to tissue tracer transport (*K*_1_), and vascular-bound tracer (*K*_*b*_) for individual participants. Crosses represent healthy controls (HC). Filled and hollow circles represent individuals in the SVD group with (WMH+) or without (WMH–) a history of lacunar stroke. Horizontal line: mean. *p < 0.05 and ***p < 0.001.

[^11^C]PBR28 *V*_*T*_ was lower in WMH than in NAWM [*t*(10) = 5.76, *p* < 0.001]. Similarly, the blood volume fraction (*V*_*b*_) was also reduced in WMH compared with NAWM [*t*(10) = 6.39, *p* < 0.001]. The plasma-to-tissue tracer transport kinetic constant (*K*_1_) was also reduced in WMH [*t*(10) = 8.29, *p* < 0.001]. In contrast, vascular TSPO binding (*K*_*b*_) was higher in WMH than in NAWM [*t*(10) = −3.24, *p* < 0.01]. There was no evidence that TSPO binding differed between symptomatic and asymptomatic individuals. Reduction in *V*_*b*_ and *K*_1_, as well as increase in *K*_*b*_ were confirmed when WHM was compared to WM tissues from healthy controls [*V*_*b*_ *t*(20) = 5.04, *p* < 0.001; *K*_1_ *t*(20) = 2.29, *p* = 0.033; *K*_*b*_ *t*_(15.3)_ = 2.52, *p* = 0.023, equal variances not assumed]. Individual data points are shown in Figure 2.

### Regional CBF Analysis

Regional CBF was reduced in WMH vs. NAWM [mean± SD 31.4± 3.8 vs. 43.0± 4.5 mL/100 g/min, *t*(8) = 16.07, *p* < 0.001, Supplementary Figure 3]. There were no significant correlations between rCBF and any of the kinetic parameters in either WMH or NAWM (Pearson's |*r*| ≤ 0.55, *p* ≥ 0.12).

### Neuropathological Assessment

**Figure 3:**
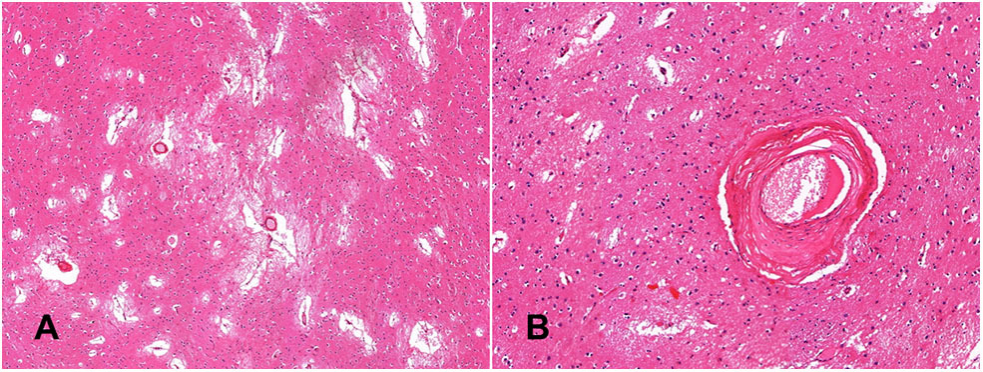
Histological confirmation of small vessel disease. The globus pallidus shows widening of perivascular spaces, loose-texture neuropil and white matter demonstrates florid reactive astrocytosis (**A**, hematoxylin-eosin—x4); perforating arteries demonstrate thickened walls; the tunica media is replaced by fibrous connective tissue (**B**, hematoxylin-eosin—x20).

**Figure 4:**
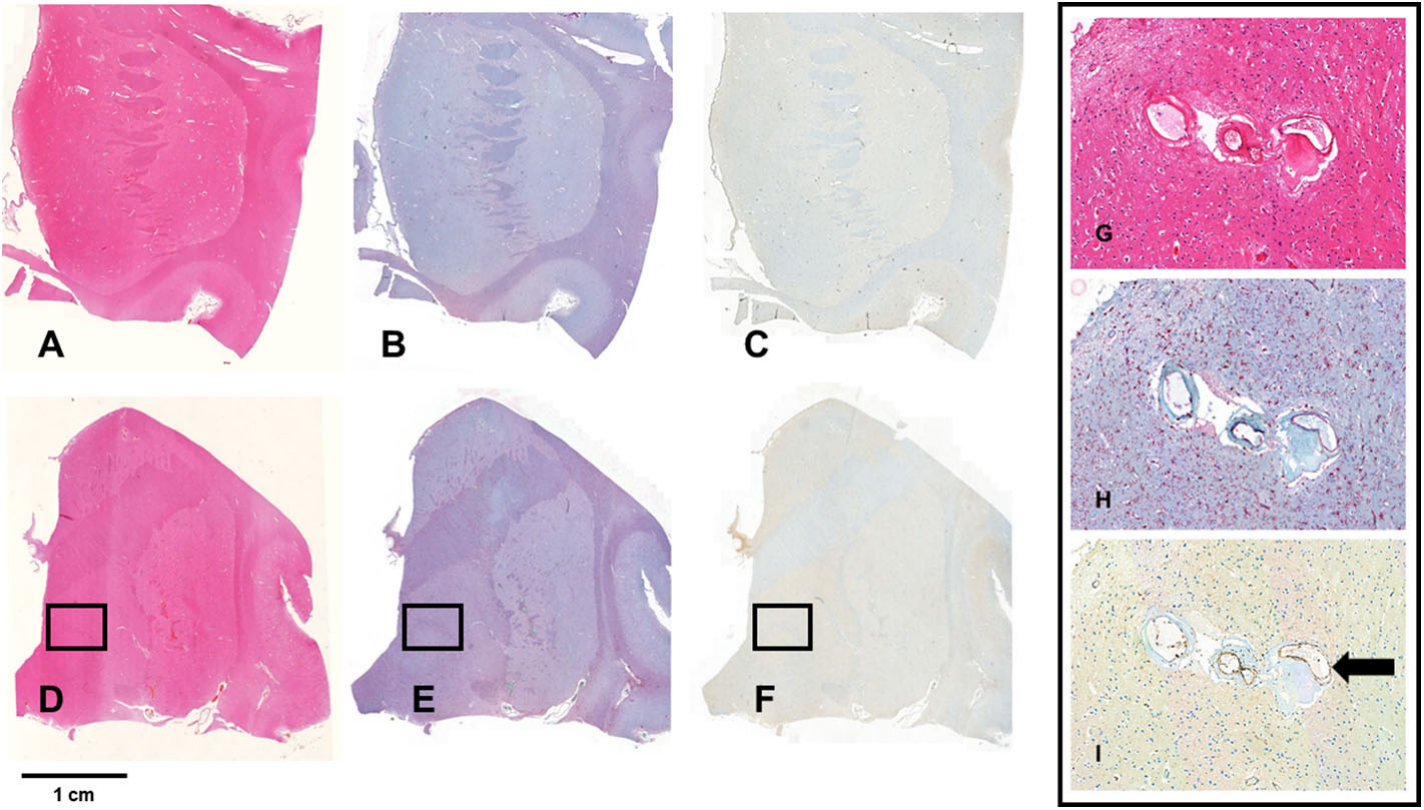
Staining for Iba1 and TSPO. **(A–F)** Show whole mount sections from the anterior **(A–C)** and posterior basal ganglia **(D,E)** from a brain with severe SVD. The sections are stained with hematoxylin-eosin **(A,D)**, and with immunochemistry for Iba1 **(B,E)** and TSPO **(C,F)**. The bar indicates 1 cm. The framed areas show the inner segment of the globus pallidus. Pictures G-I show a x20 magnification of the framed area. Perforating arteries have thickened walls (**G**, HE—x20); there is florid microglial and macrophagic response (red cells) (**H**, Iba1 immunostain—x20); TSPO expression is low and limited to a minority of microglia cells. In contrast, endothelial cells are intensely positive (arrow) (**I**, TSPO immunostain—x20).

**Figure 5:**
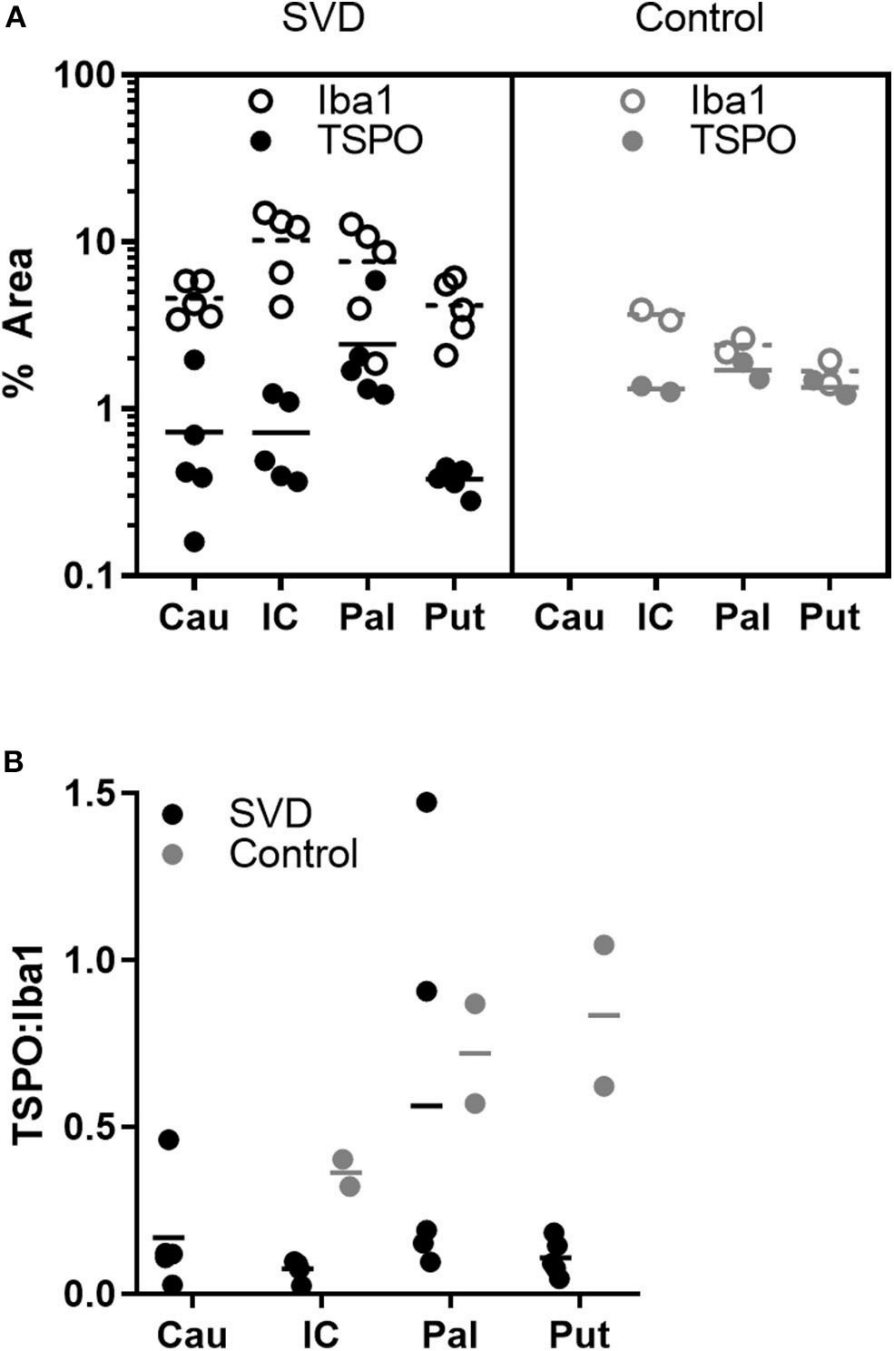
Fewer microglia express TSPO in SVD than control tissue. **(A)** Individual measurements of the pan-microglial marker Iba1 (hollow markers) and TSPO (filled markers) in SVD and healthy controls. In SVD, there are greater numbers of microglia but a smaller proportion express TSPO; **(B)** ratios of TSPO:Iba1 density in each group. The ratio is significantly lower in SVD over all regions. Horizontal line = mean.

The five *post-mortem* cases showed features of severe SVD according to the criteria proposed by Skrobot et al. (31) (Figure 3). One of the brains showed a microinfarct in the anterior putamen that was excluded from tissue sampling. The distribution and density of microglial cells in striatum and anterior and posterior limbs of the internal capsule was similar across the five cases. Of microglia positively stained with Iba1, a mean of only 23% also stained positively for TSPO (Table 2, Figure 4). The fraction of TSPO-positive microglia was similar in all regions examined. The breakdown of values in each region is shown in Table 2 and individual values are presented in Figure 5. TSPO was expressed in the endothelium as normally seen in vessels whereas expression was considerably reduced or absent in the tunica media of vessels with fibrosis of their walls. When present, perivascular macrophages were TSPO-positive whereas no detectable TSPO expression was present in perivascular astrocytes.

**Table 2:** Immunohistochemical measures.

**Group**	**ROI**	**Iba1 density**	**TSPO density**	**Density ratio (%)**
SVD	Caudate	4.60	0.73	17
	Internal Capsule	10.21	0.72	8
	Pallidus	7.62	2.43	56
	Putamen	4.15	0.38	11
	**Mean**	**6.65**	**1.07**	**23**
HC	Internal Capsule	3.66	1.24	34
	Pallidus	2.41	1.70	72
	Putamen	1.68	1.35	83
	**Mean**	**2.58**	**1.43**	**63**

Perforating arteries in the two control brains showed thin walls, only minimal widening of perivascular spaces and mild perivascular astrocytosis. The mean proportion of Iba1 stained microglia also staining positively for TSPO was 63%. The ratio of TSPO to Iba1 density differed significantly between groups [*F*(1,15) = 5.79, *p* = 0.029] but not regions [*F*(2,15) = 2.49, *p* = 0.116]. This effect appeared to be driven by an increase in Iba1 density in SVD tissue [*F*(1,15) = 7.93, *p* = 0.013], while TSPO density did not differ between groups [*F*(1,15) = 0.25, *p* = 0.623].

For comparison with the neuropathological assessment, we modeled PET TSPO tracer binding in the striatum. There were no significant differences between participants with SVD and healthy controls in the striatum with either the 2TCM or the 2TCM-1K model. Of the 11 participants with SVD, only three had one or more lesions >4 mm in the striatum, with one accompanied by widespread smaller lesions. These three participants are distinguished in Figure 2 and do not appear to differ from the rest of the SVD group.

## Discussion

Expression of TSPO, often increased in the presence of activated microglia (32), was reduced in WMH in comparison with normal-appearing white matter. In contrast, TSPO binding in the vascular compartment was higher in WMH, relative to both normal-appearing tissue in SVD and white matter of healthy individuals free of SVD. Immunohistochemistry in *post-mortem* brain tissue showed a higher number of Iba1-positive microglia in SVD, but a reduction in the proportion of TSPO-positive microglia. In and around affected small vessels, TSPO expression was found in vessel walls and perivascular macrophages, consistent with the PET binding results. These results suggest an alteration of the phenotype of activated microglia in ischemic WMH, in which microglial activation is uncoupled from TSPO expression. Furthermore, they suggest that TSPO can provide potentially useful information about vascular and perivascular pathology in SVD.

A previous *post-mortem* analysis of microglial staining, with careful alignment of histological sampling and imaging abnormalities, was performed in the MRC-CFAS neuropathology cohort (9). Activated microglia were found to be abundant in regions affected by WMH and present at lesser abundance in normal-appearing white matter. However, in the MRC-CFAS study, an activated microglial phenotype was inferred from expression of HLA-DR; immunohistochemistry of TSPO was not performed. In the present study, histological analysis of post-mortem white matter confirmed that microglia are abundant in SVD (Figure 4). However, many of these microglia stained negatively for TSPO (Figure 5). One possible explanation is that the microglial phenotype—and particularly the upregulation of mitochondrial biosynthesis—is altered in the ischemic conditions of visible areas of injury in SVD. Relative ischemia, compared with normal-appearing white matter, was confirmed experimentally in WMH in the patients investigated with PET. Conceivably, microglial activation without enhanced expression of TSPO occurs because regions of WMH are chronically hypoperfused, so that the role of oxidative phosphorylation, and thereby mitochondrial proliferation, in the microglial response is diminished. Alternatively, microglial phenotype may change as tissue injury and repair enters a more chronic phase (i.e., in contrast to acute ischemic injury). Little is known about the role of activated microglia at a late stage remote from injury. Persistent activated morphology could reflect either ongoing tissue remodeling or recurrent immune challenge, for example from cells or molecules that cross a compromised blood-brain barrier. If there is a *chronic* activated microglial phenotype, this might be less tightly coupled to mitochondrial biosynthesis and metabolism. Altered tracer dynamics might have also influenced the pattern of results. The decreases in the modeled tracer transport suggest exactly this. However, even after modeling these effects, there is still an apparent reduction in TSPO binding. Overall, the results point not to a reduction of inflammatory response in WMH, but rather to an altered phenotype of activated microglia, with reduced TSPO expression.

We found no evidence that the pattern of TSPO binding was different in individuals with symptomatic stroke in the year leading up to PET. The number of individuals in each subgroup was small so these results must be interpreted with caution. However, the lack of an obvious difference in the scatter plots in Figure 2 argues against the contention that neuroinflammation is limited to those with recent symptoms.

Our findings would fit with a view of progression of SVD whereby initial increases in TSPO expression are followed by a chronic phase in which decreased TSPO accompanies worsening hypoperfusion and increased damage to neurons. Imaging studies have shown that leakage of plasma proteins begins early in SVD and creates an inflammatory microenvironment that sustains and maintains tissue damage (33). Macrophages and activated microglia release proteases, reactive oxygen species (ROS) and reactive nitrogen species that can attack the blood vessel walls, extra cellular matrix and myelin (33, 34). Increased TSPO in microglia initially protects brain tissue from high levels of ROS (35). During the course of the disease lower TSPO in microglia and macrophages can reflect a progressive reduction of inflammatory response (36) but conversely can be instrumental in maintaining tissue damage given its role in the resistance against ROS cytotoxicity. In addition, ROS and nitric oxide intermediates produced by activated microglia are effective in damaging mitochondria and the resulting mitochondrial dysfunction can cause further downregulation of TSPO. Low microglial TSPO in SVD could therefore reflect a chronic “toxic state” in which microglia-induced ROS exceeds antioxidant defenses with subsequent injury of neurons, ECM and vessel walls (35).

Interestingly, the differences in tracer kinetic modeling parameters between WHM and NAWM were consistent with both standard 2TCM and 2TCM-1K (see Supplementary Material). The latter includes an additional term that separates TSPO tracer binding within parenchymal and vascular compartments. Modeling [^11^C]PBR28 with 2TCM-1K has shown several advantages compared to standard 2TCM. Firstly, 2TCM-1K leads to a better and more efficient data description (improved fit and lower Akaike coefficient) compared to standard 2TCM in both healthy individuals and patients with CNS diseases (18, 25). Secondly, the 2TCM-1K is more sensitive to changes in affinity as demonstrated by its higher sensitivity to changes in rs6971 polymorphism (25). Finally, the 2TMC-1K has a stronger agreement with TSPO mRNA expression than the 2TCM—this was demonstrated both in terms of binding data at baseline (25) and in terms of displacement after TSPO blocking (17). The work presented here extends existing literature by showing how 2TCM-1K can also be used to investigate TSPO distribution at the intact and disrupted vascular interface. Irrespective of the type of model used for the tracer quantification, both 2TCM and 2TCM-1K showed a reduction of blood volume (*V*_*b*_) and blood to tissue tracer transport (*K*_1_) in WHM as compared to NAWM and WM tissues in healthy controls, so that the main PET results are consistent across models.

Explicit modeling of the vascular compartment led to the striking finding of an increase in the vascular tracer binding constant, *K*_*b*_, in WMH compared to NAWM and healthy white matter. Elevated *K*_*b*_ most likely reflects a higher density of TSPO in or around vessel walls. TSPO is expressed in endothelium and the extent of WMH in patients with SVD correlates with thrombomodulin, a circulating marker of endothelial cell activation (6). However, endothelial TSPO staining appeared normal in our *post-mortem* SVD specimens. Perivascular macrophages also bind TSPO ligands and were indeed observed in our specimens. Higher [^11^C]PBR28 signal in the vascular compartment in SVD may be further explained by the formation of pockets delimited by collagen type IV-positive membranes in vessels walls (37). These pockets contain plasma proteins which can bind and entrap TSPO ligands, delaying their diffusion through vessel walls (38, 39). According to this hypothesis, the increase in TSPO PET signal in the vascular compartment in SVD may be driven in party by tracer trapped within the vessel wall rather than a true increase in TSPO expression.

The persistence of TSPO tracer in vascular compartment might also reflect the progressive loss of smooth muscle cells in the tunica media and their replacement by fibrous connective tissue, collagen type IV in particular (17), with a possible subsequent increase in resistance to lipophilic tracers.

In addition to offering new insight into the pathogenesis of SVD, the current results have important implications for the design and interpretation of PET studies that utilize TSPO as a marker of neuroinflammation. Given the fact that in white matter lesions the TSPO signal was mainly at the interface between brain parenchyma and vascular unit, blood sampling and full compartmental modeling is fundamental to distinguish the contribution of different compartments to the measured PET signal. At the same time, reference region quantification approaches are not likely to be appropriate because of the assumption of a similar blood-to-tissue tracer exchange between target and reference tissue. Therefore, in participants with evidence of cerebrovascular disease, TSPO PET studies should adopt blood sampling and full compartment modeling approaches and avoid analyses that depend on reference regions. Practical considerations often argue against invasive blood sampling in older groups; the present results show that full modeling strategies are most needed in groups who may find these procedures more difficult to tolerate. The third implication is that TSPO upregulation and microglial proliferation are uncoupled in damaged tissue: TSPO cellular dynamics are more complex than a simple “TSPO upregulation equals microglia activation.” The presence of damaged white matter is not confined to those with prior lacunar stroke or a diagnosis of SVD, so these implications extend to other clinical settings. A large proportion of patients with established AD, perhaps as many as 40%, have diffuse WMH. The possibility of activated but TSPO-negative microglia in areas of WMH will require a more careful interpretation of TSPO PET binding results in a wide range of clinical settings.

The interpretation of TSPO PET signal as a true marker of microglial activation and therefore neuroinflammation has been a matter of debate in molecular imaging studies (40, 41). Indeed, TSPO signal can be driven by other factors, such as recruitment of peripheral monocytes into the parenchyma, adherence of circulating leucocytes to the vascular endothelium and the expression of TSPO in other CNS cells including astrocytes, vascular endothelial cells and neurons. Potential avenues for future studies might include use of dual tracers with different properties in terms of cell type or compartment specificity. The development of new tracers with specificity for different classes of immune cells or cell surface markers would also be a major advance. The current results add the possibility of TSPO-negative activated microglia to the list of provisos and underscore the value of parallel PET and *post-mortem* analyses.

### Strengths and Limitations

This study has limitations. The sample sizes for both the PET and *post-mortem* studies are small. This study used a full PET design with dynamic acquisition and arterial blood sampling using high-specific TSPO tracer as [^11^C]PBR28. Arterial blood sampling is invasive, which limits its use with participants who are elderly or ill. The intention was to provide a preliminary study adopting a comprehensive PET methodology, which could be used to plan larger studies and provide a starting point to explore less invasive methods, which have been developed (42–44) but require more validation work before extending them to SVD disease. The healthy control participants were younger than the group with SVD, on average. Several TSPO studies show a positive association between age and TSPO brain expression (45–47), raising a confounding effect of age on the differences we report between the SVD and healthy controls (see Supplementary Figure 4). However, a recent study on a large sample (*N* = 140) of [^11^C]PBR28 PET scans shows that this effect might be limited to cortical regions only (48). Measurement of cerebral blood flow with arterial spin labeling made it possible to explore the relationships between tissue blood flow and TSPO binding. However, for both methods, signal-to-noise ratio is relatively low in white matter, limiting the precision with which these associations can be explored. The PET regions of interest were matched as closely as possible to the anatomical landmarks used for post-mortem tissue sampling, but a fixed frame of reference based on post-mortem MRI was not available for this study. A future study would also be strengthened by obtaining systemic markers of inflammation from individuals undergoing PET.

### Conclusion

Elevated TSPO binding provides evidence of inflammatory activation localized to vessel walls and perivascular spaces in SVD. Reduced TSPO expression despite microglial proliferation and tissue pathology consistent with inflammation may reflect mitochondrial deficiency in microglia as a result of chronic hypoxia or chronic oxidative stress. Our findings add further evidence for a pivotal role of the neurovascular unit in the pathogenesis of SVD and should prompt extra caution when interpreting TSPO PET in older individuals or those with vascular risk.

## Data Availability Statement

The data generated for this study are available upon appropriate request, addressed to the corresponding author.

## Ethics Statement

The studies involving human participants were reviewed and approved by London Bromley Research Ethics Committee (reference 13-LO-1745). Participants provided informed, written consent to participate in this study.

## Author Contributions

PW recruited participants, acquired and processed MR data, analyzed MR and PET statistics, and contributed to the paper. MV analyzed PET data and contributed to the paper. NM recruited participants and acquired PET data. FT, ER, CB, and SW assisted in the design of the experiments, interpretation of the data, and preparation of the manuscript. AH selected, immunostained, analyzed, and quantified normal control brains. BO and RW performed tissue processing, immunostaining, and quantification of Iba1 and TSPO in SVD brains. TM and OH contributed PET data from healthy controls. FR designed the methodology of tissue analysis, selected the cases, and contributed to manuscript writing. MO'S conceived the study, designed and oversaw the PET and MRI studies, and contributed to the paper. All authors contributed to the article and approved the submitted version.

## Conflict of Interest

ER is employed by Invicro (UK). The remaining authors declare that the research was conducted in the absence of any commercial or financial relationships that could be construed as a potential conflict of interest.
