# Supplementary material for: Patterns of Mitochondrial TSPO Binding in Cerebral Small Vessel Disease: An in vivo PET Study With Neuropathological Comparison
Source: Front Neurol. 2020 Oct 16;11:541377. doi: 10.3389/fneur.2020.541377 (PMC7596201; doi:10.3389/fneur.2020.541377)
Supplement: Supplementary file 1 [file Table_1.docx]

Supplementary Material

# Macros for imageJ

Two separate automated counting macros coded in JavaScript were run on ImageJ to count the percentage of area occupied by positive staining signal of TSPO or Iba1 in each ROI. Each image was separated into the three Q22 RGB channels by applying the Color Deconvolution tool using the H DAB vector. The red channel, showing the oxidized DAB brown precipitate indicating positive immunohistochemistry staining for TSPO or Iba1, was selected and converted to a binary image. The threshold was adjusted to minimize background staining artifacts and applied consistently across each ROI. The percentage area occupied by the positively stained signal indicated by black was calculated with the Analyze Particle tool.

## Macro for TSPO

imageTitle=getTitle();//returns a string with the image title

run("Colour Deconvolution", "vectors=[H DAB]");

selectWindow(imageTitle);

close(imageTitle);

close(imageTitle+"-(Colour_1)");

close(imageTitle+"-(Colour_3)");

run("Sharpen");

run("8-bit");

setAutoThreshold("Default");

run("Threshold...");

setThreshold(0, 164);

setOption("BlackBackground", false);

run("Convert to Mask");

run("Close");

run("Make Binary");

run("Watershed");

run("Analyze Particles...", "size=20-Infinity circularity=0.00-4.00 show=Outlines clear summarize");

## Macro for Iba1

imageTitle=getTitle();//returns a string with the image title

run("Colour Deconvolution", "vectors=[H DAB]");

selectWindow(imageTitle);

close(imageTitle);

close(imageTitle+"-(Colour_1)");

close(imageTitle+"-(Colour_3)");

run("Sharpen");

run("8-bit");

setAutoThreshold("Default");

run("Threshold...");

setThreshold(0, 161);

setOption("BlackBackground", false);

run("Convert to Mask");

setThreshold(255, 255);

run("Close");

setThreshold(255, 255);

run("Make Binary", "thresholded remaining black");

run("Watershed");

run("Analyze Particles...", "size=20-Infinity circularity=0.00-4.00 show=Outlines clear summarize");

# Supplementary Figures


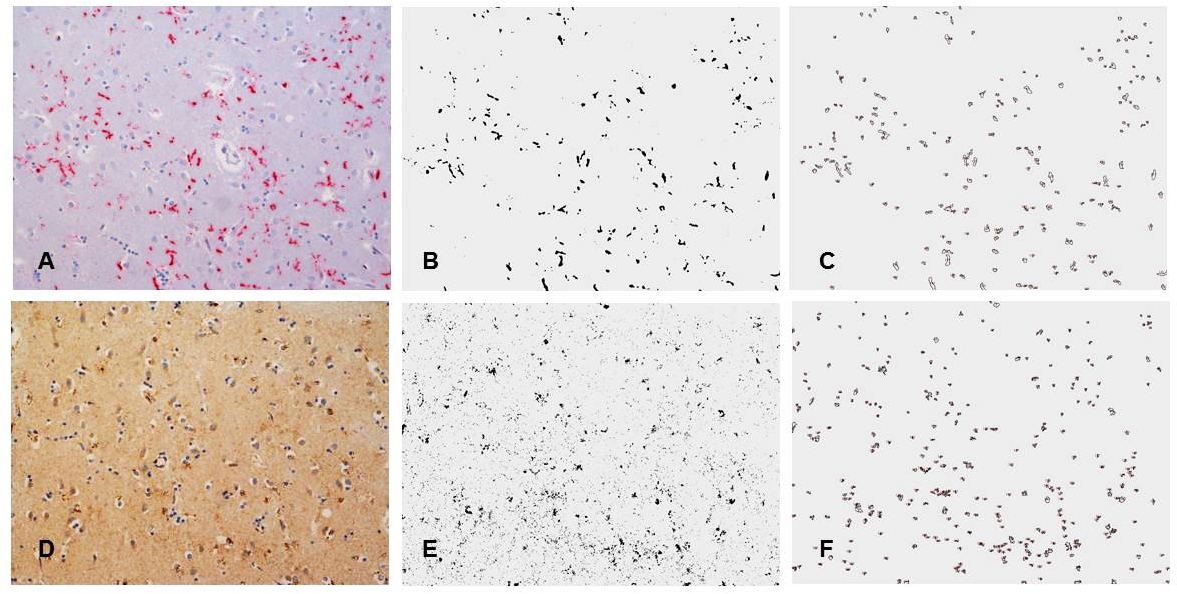


**Supplementary Figure 1:** This panel represents the quantification of microglial density and TSPO expression in a control brain: Image A and D show Iba1 and TSPO immunohistochemistry respectively (x20); binary black and white images after adjusting to minimise background staining artefacts are shown in images B and E. The background is cleaned with Analyse Particle Tool leaving positive cells to be measured (images C and F).


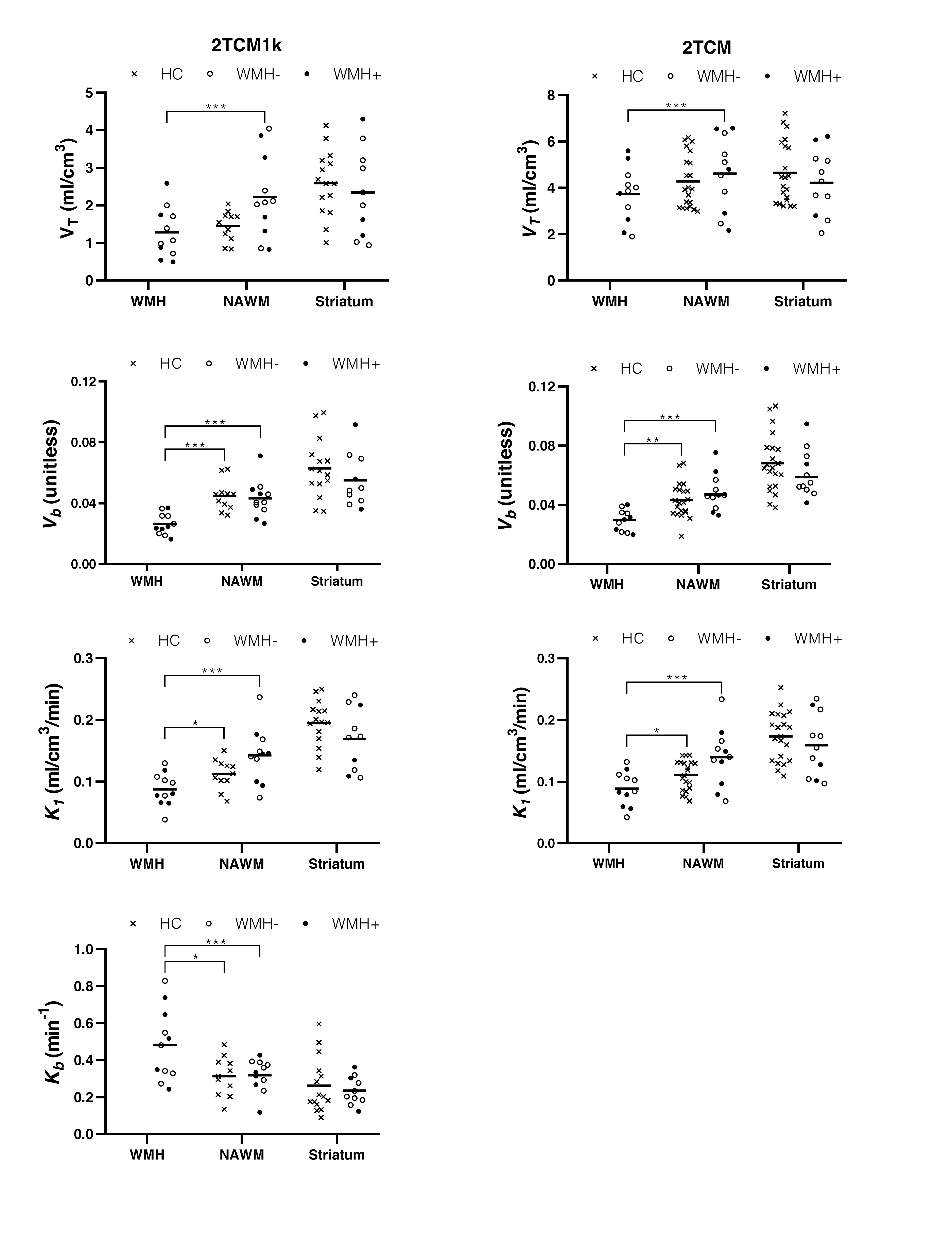


**Supplementary Figure 2.** TSPO binding parameters differ between normal appearing white matter (NAWM) and white matter hyperintensities (WMH). Similar results are seen with the 2-tissue compartmental model with the inclusion of vascular binding (2TCM-1K; left column) and the standard 2-tissue compartmental model (2TCM; right column). Binding parameters for NAWM also differed between healthy controls and participants with WMH. Plots show volume of tracer (*V_T_*), tissue-to-blood ratio (*V_b_*), plasma to tissue tracer transport (*K_1_*) and vascular-bound tracer (*K_b_*) for individual participants. Crosses represent healthy controls (HC). Filled and hollow circles represent individuals in the SVD group with (WMH+) or without (WMH-) a history of lacunar stroke, either in the whole brain for WMH and NAWM ROIs or in the striatum for the striatum ROI. Horizontal line: mean. * p < .05. *** p < .001.


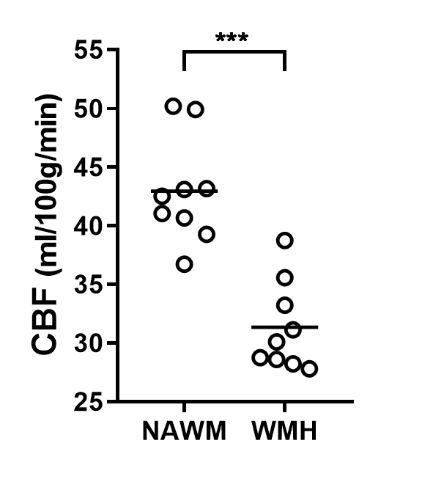


**Supplementary Figure 3: Cerebral blood flow is reduced in WMH.** NAWM: normal-appearing white matter. WMH: white matter hyperintensities. Horizontal line = mean.

# Supplementary Tables

**Supplementary Table 1.** Paired t-tests comparing WMH and NAWM ROIs in SVD patients.

|  | **2TCM-1K** | | **2TCM** | |
| --- | --- | --- | --- | --- |
| **Parameter** | **t(10)** | **p** | **t(10)** | **p** |
| *V_T_* | 5.76 | 0.00018 | 5.54 | 0.00025 |
| *V_b_* | 6.39 | 0.00008 | 6.79 | 0.00005 |
| *K_1_* | 8.29 | 0.00001 | 7.65 | 0.00002 |
| *K_b_* | -3.67 | 0.00433 | n/a |  |

**Supplementary Table 2.** Differences between SVD patients and healthy controls.

|  |  | **2TCM1k** | | |  | **2TCM** | | |
| --- | --- | --- | --- | --- | --- | --- | --- | --- |
|  | **Parameter** | **t** | **df** | **p** |  | **t** | **df** | **p** |
| Striatum | *V_T_* | 0.52 | 24.0 | .605 |  | 0.86 | 30 | .398 |
|  | *V_b_* | 0.88 | 24.0 | .388 |  | 1.01 | 30 | .320 |
|  | *K_1_* | 1.26 | 24.0 | .221 |  | 0.89 | 30 | .379 |
|  | *K_b_* | 0.52 | 23.0 | .610 |  | n/a |  |  |
| WMH vs HC NAWM | *V_T_* | 0.71 | 20.0 | .483 |  | 1.29 | 30 | .208 |
|  | *V_b_* | 5.04 | 20.0 | <.001 |  | 3.52 | 30 | .001 |
|  | *K_1_* | 2.29 | 20.0 | .033 |  | 2.16 | 30 | .039 |
|  | *K_b_* | -2.52 | 15.3 | .023 |  | n/a |  |  |

WMH: white matter hyperintensities. HC: healthy controls. NAWM: normal-appearing white matter.
